# Different acupuncture intervention time-points for improving capacity in motor function and activities of daily living after stroke: A protocol for systematic review and network meta-analysis

**DOI:** 10.1097/MD.0000000000024578

**Published:** 2021-02-05

**Authors:** Yue Zhuo, Shifeng Deng, Ming Xu, Yuchen Zhang, Xiaoye Lu, Boyu Wu, Hong Zhang

**Affiliations:** College of Acupuncture-Moxibustion and Tuina, Hunan University of Chinese Medicine, Changsha, Hunan, China.

**Keywords:** acupuncture, network meta-analysis, protocol, stroke, systematic review

## Abstract

**Background:**

The incidence of stroke has been found in an increasing trend worldwide, resulting in significant negative effects and severe impairments to survivors in terms of motor function and activities of daily living. Acupuncture therapy has been widely used in the clinical treatment of stroke for a long time, meanwhile, the efficacy has been confirmed by many studies. However, the optimal intervention time-point of acupuncture in stroke is controversial. Therefore, the purpose of our study is to provide scientific evidence and reasonable suggestions for this issue.

**Methods:**

A computer-based retrieval will be employed in 7 electronic databases: EMBASE Database, PubMed, the Cochrane Library, China National Knowledge Infrastructure (CNKI), Wan Fang databases, Chinese Scientific Journals Database (VIP) and China Biological Medicine Database (CBM), from the establishment date of each database throughout October 2020. Only randomized controlled trials of acupuncture for stroke will be recruited and language is limited to English or Chinese. The outcomes we focus on include the Fugl-Meyer Assessment score and the Barthel Index. Additionally, safety assessments such as adverse events and drop-out cases may also be taken into consideration. The network meta-analysis will be performed based on the Bayesian framework and literature selection will be conducted by 2 trained reviewers. All data analysis will be calculated by Revman5.3, WinBUGS 1.4.3, Stata13.0, and R software 3.6.1. The Assessment of heterogeneity, inconsistency, subgroup, sensitivity, and publication bias will also be done under the guidelines of Cochrane Collaboration's tool.

**Results:**

The results of this study will be submitted to a peer-reviewed journal for publication.

**Conclusion:**

This network meta-analysis will provide evidence-based references to evaluate the efficacy of different acupuncture intervention time-points during the treatment of stroke. Furthermore, it will help the clinicians to formulate appropriate medical plans and improve clinical efficacy.

**Trials registration number:**

INPLASY2020120060

## 1 Introduction

Stroke is a common disease that endangers the physical and mental health of human beings.^[1]^ In China, stroke is the first cause of death and due to an aging population, its incidence is increasing, meanwhile, it has been reported that stroke is the second leading cause of death and a major cause of disability around the world.^[2,3]^ Post-stroke disability can include impairments in motor, sensory, cognitive functions, and so on, all the above severely compromise the activities of daily living in patients after stroke. It is not hard to understand that stroke finally results in a huge burden on health care and financial situation to their own families as well as the whole society.^[4]^

With the development of medical sciences and iatrical technologies, significant advancement has been made in the diagnosis, prevention, and treatment of cerebral stroke. The routine treatment methods for stroke contain surgical interventions, pharmaceuticals, symptomatic, and supportive measures as well as rehabilitation therapy.^[5]^ However, concerns about these approaches also have been aroused such as high costs of treatments, side effects of the drugs, or the invasive procedure of the surgery, more importantly, the sequelae of the disease.^[6]^ Because of the individual difference in disease severity or clinical symptoms and the gap of medical conditions in diverse regions, post-stroke motor dysfunction and impairment in the activities of daily living still maintains a great incidence, which reminds a challenging problem to be solved.^[7]^

Acupuncture is an ancient Chinese medicine treatment, which has a wide range of clinical applications. In recent years, more and more attention has been paid to acupuncture therapy in dealing with stroke.^[8–11]^ It has been proved that acupuncture has a great effect on the improvement of limb motor function as well as the activities of daily life for the patients after stroke.^[12–15]^ Its mechanism may be related to the promotion of neurogenesis and cell proliferation in the lesion area and the regulation of neurochemicals.^[16–18]^ However, the quality of these researches varies.^[19–21]^ Moreover, there is a lack of studies on what is the optimal acupuncture intervention time-point for patients with stroke. In other words, to maximize the benefits of patients, which stage of stroke should acupuncture be adopted in? There has been no clear conclusion by now. Therefore, there is an urgent need for medical practitioners and scientific researchers to explore further and summarize more.

At present, there are many randomized controlled trials (RCTs) of acupuncture therapy on stroke-related diseases, but the acupuncture intervention time-point of stroke is different, including hyperacute stage, acute stage, subacute stage, convalescent stage, and sequela stage, accordingly, the outcomes are varying.^[22–25]^ Thus, it is important for us to find out what is the optimal acupuncture intervention time-points for patients after stroke in terms of the improvement of motor function and activities of daily living. It can not only help these patients recover better, but also save more medical resources. Obviously, it is impossible to answer this question well through traditional meta-analysis, however, using the way of Bayesian network meta-analysis can compare the therapeutic effect of multiple acupuncture intervention time-points. In our research, the efficacy of different acupuncture intervention time-point on post-stroke motor dysfunction and activities of daily living will be ranked by network meta-analysis, what is more, to provide reasonable and scientific evidence-based medicine basis for the clinical participants to refer.

## 2 Methods

### 2.1 Protocol and registration

This protocol for systematic review and network meta-analysis will strictly comply with the preferred reporting items for systematic review and meta-analysis protocols (PRISMA-P) guidelines.^[26]^ Meanwhile, the ultimate report will abide by the recommendations of the PRISMA Extension Statement for reporting of systematic reviews incorporating network meta-analyses of health care interventions.^[27]^ We have acquired the registration number(INPLASY2020120060; DOI:10.37766/inplasy2020.12.0060) of this study on the INPLASY website. We will update the detailed information in the final report in time if there are any modifications during the whole research period.

### 2.2 Ethics

Given that the systematic review and network meta-analysis does not involve the collection of private information, ethical approval is not required of this study. However, giving an informed consent form to every patient before treatment is necessary.

### 2.3 Inclusion and exclusion criteria

Our inclusion and exclusion criteria were established by the principle of “PICOS” (participants, intervention, comparator, outcomes, and study designs).

#### 2.3.1 Types of studies

Only RCTs of acupuncture for stroke will be recruited and regardless of population characteristics, blind method, and duration of trials. However, the language is limited to English or Chinese. We will remove non-RCTs such as meeting abstracts, clinical experience, case reports, system reviews, animal trails, duplications meeting abstracts. Additionally, Studies should be available in full papers as well as peer-reviewed and the original data should be clear and sufficient.

#### 2.3.2 Types of participants

All adults with a recent or past medical history of ischemic or hemorrhagic stroke, which is diagnosed with clearly defined or internationally recognized criteria (e.g., confirmed by CT or MRI scan) will be eligible for recruitment and regardless of nationality, race, gender, age, educational background. However, the patients who are not medically stable or unable to follow basic commands will be excluded.

#### 2.3.3 Types of interventions and comparators

The control group takes non-acupuncture treatment, including rehabilitation treatment, or combined with symptomatic and supportive treatment. The treatment of the experimental group is on basis of the control group, besides this, acupuncture or one of the following related treatment (acupoint-based therapy): electroacupuncture, auricular acupuncture, head acupuncture, warm acupuncture, hand acupuncture must be used in the experimental group. Participants in both groups could receive routine medical treatment and regardless of treatment duration, as well as frequency.

#### 2.3.4 Types of outcomes

The primary outcome is measured with the Fugl-Meyer Assessment score, which has been widely used to evaluate the limb motor function of patients after stroke.^[28,29]^ The secondary outcome includes the Barthel Index, which can assess the activities of daily living accurately.^[30]^ Additionally, safety assessments such as adverse events and drop-out cases may also be taken into consideration.

### 2.4 Database and search strategy

An all-round online search for published related studies will be conducted in the following academic databases from their inception throughout October 2020: EMBASE Database, PubMed, the Cochrane Library, China National Knowledge Infrastructure (CNKI), Wan Fang databases, Chinese Scientific Journals Database (VIP) and China Biological Medicine Database (CBM). The MeSH terms together with free words will be adopted as the basic strategy of the search and the language is limited to English or Chinese. Additionally, conference literature and relevant references will also be checked carefully under the guidelines of the snowball strategy.^[31]^ If necessary, we will try to contact the corresponding author to get the information we need. The initial retrieval strategy of PubMed is shown in Table 1 as an example.

**Table 1:** PubMed search strategy draft.

Number	Search items
#1	“Stroke”[mesh]
#2	“Cerebral hemorrhage”[mesh]
#3	Strokes[title/abstract] or cerebrovascular accident[title/abstract] or cerebrovascular accidents[title/abstract] or cva[title/abstract] or cvas[title/abstract] or cerebrovascular apoplexy[title/abstract] or apoplexy, cerebrovascular[title/abstract] or vascular accident, brain[title/abstract] or brain vascular accident[title/abstract] or brain vascular accidents[title/abstract] or vascular accidents, brain[title/abstract] or cerebrovascular stroke[title/abstract] or cerebrovascular strokes[title/abstract] or stroke, cerebrovascular[title/abstract] or strokes, cerebrovascular[title/abstract] or apoplexy[title/abstract] or cerebral stroke[title/abstract] or cerebral strokes[title/abstract] or stroke, cerebral[title/abstract] or strokes, cerebral[title/abstract] or stroke, acute[title/abstract] or acute stroke[title/abstract] or acute strokes[title/abstract] or strokes, acute[title/abstract] or cerebrovascular accident, acute[title/abstract] or acute cerebrovascular accident[title/abstract] or acute cerebrovascular accidents[title/abstract] or cerebrovascular accidents, acute[title/abstract]
#4	Hemorrhage, cerebrum[title/abstract] or cerebrum hemorrhage[title/abstract] or cerebrum hemorrhages[title/abstract] or hemorrhages, cerebrum[title/abstract] or cerebral parenchymal hemorrhage[title/abstract] or cerebral parenchymal hemorrhages[title/abstract] or hemorrhage, cerebral parenchymal[title/abstract] or hemorrhages, cerebral parenchymal[title/abstract] or parenchymal hemorrhage, cerebral[title/abstract] or parenchymal hemorrhages, cerebral[title/abstract] or intracerebral hemorrhage[title/abstract] or hemorrhage, intracerebral[title/abstract] or hemorrhages, intracerebral[title/abstract] or intracerebral hemorrhages[title/abstract] or hemorrhage, cerebral[title/abstract] or cerebral hemorrhages[title/abstract] or hemorrhages, cerebral[title/abstract] or brain hemorrhage, cerebral[title/abstract] or brain hemorrhages, cerebral[title/abstract] or cerebral brain hemorrhage[title/abstract] or cerebral brain hemorrhages[title/abstract] or hemorrhage, cerebral brain[title/abstract] or hemorrhages, cerebral brain[title/abstract]
#5	#1 or #2 or #3 or #4
#6	“Acupuncture”[mesh]
#7	“Acupuncture points”[mesh]
#8	“Acupuncture, ear”[mesh]
#9	“Acupuncture analgesia”[mesh]
#10	“Acupuncture therapy”[mesh]
#11	“Auriculotherapy”[mesh]
#12	Acupuncture[title/abstract] or acustimulation[title/abstract] or triggerpoint[title/abstract] or acupuncture analgesia[title/abstract] or silver needle[title/abstract] or moxibustion[title/abstract] or de qi[title/abstract] or electro-acupuncture[title/abstract] or meridian[title/abstract] or auriculotherapy[title/abstract] or extra points[title/abstract] or needle pricking[title/abstract] or transcutaneous electric nerve stimulation[title/abstract] or acupressure[title/abstract] or needling[title/abstract] or intradermal needle[title/abstract] or point application[title/abstract] or fire needle[title/abstract] or three-edged needle[title/abstract] or a-shi point[title/abstract] or five phase points[title/abstract] or needle-embedding[title/abstract] or pricking therapy[title/abstract] or point injection[title/abstract] or incision therapy[title/abstract] or pharmacopuncture[title/abstract] or acupuncture treatment[title/abstract] or acupuncture treatments[title/abstract] or treatment, acupuncture[title/abstract] or therapy, acupuncture[title/abstract] or pharmacoacupuncture treatment[title/abstract] or treatment, pharmacoacupuncture[title/abstract] or pharmacoacupuncture therapy[title/abstract] or therapy, pharmacoacupuncture[title/abstract] or acupotomy[title/abstract] or acupotomies[title/abstract] or acupuncture point[title/abstract] or point, acupuncture[title/abstract] or points, acupuncture[title/abstract] or acupoints[title/abstract] or acupoint[title/abstract]
#13	#6 or #7 or #8 or #9 or #10 or #11 or #12
#14	“Randomized controlled trial” [publication type]
#15	“Randomized controlled trials as topic”[mesh]
#16	“Pragmatic clinical trial” [publication type]
#17	“Pragmatic clinical trials as topic”[mesh]
#18	“Intention to treat analysis”[mesh]
#19	“Random allocation”[mesh terms]
#20	Random∗[title/abstract]
#21	#14 or #15 or #16 or #17 or #18 or #19 or #20
#22	#5 and #13 and #21

### 2.5 Literature selection and data extraction

**Figure 1:**
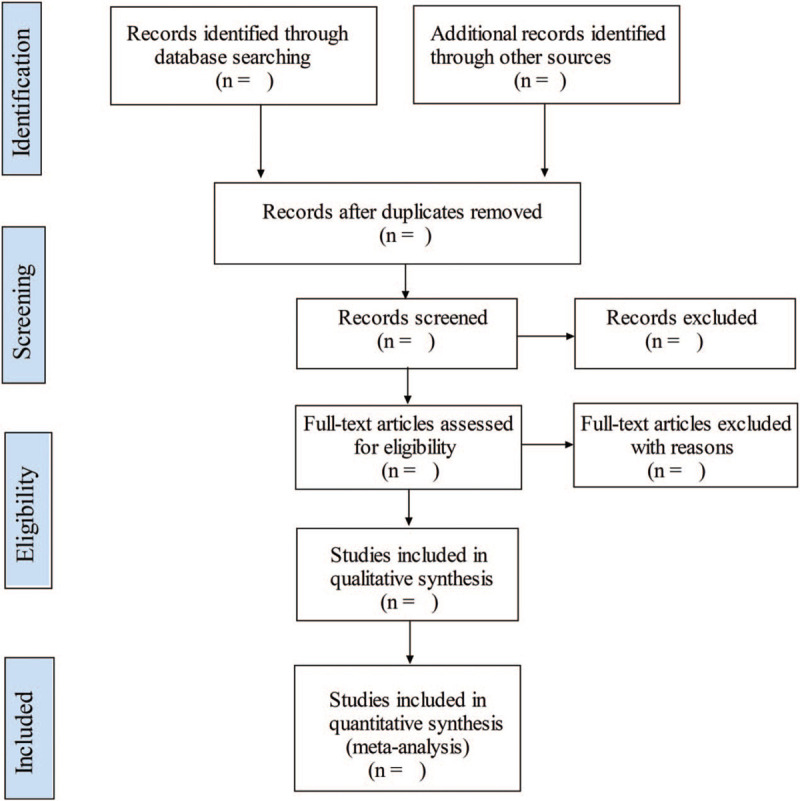
Flow chart of study selection.

Firstly, the literature retrieved from the above databases will be imported into the EndNoteX7 tool for preliminary screening, then duplicate articles will be removed. Secondly, 2 independent reviewers (Yue Zhuo and Boyu Wu) will glance at the titles and abstracts, then studies obviously not meeting the inclusion criteria will be removed. Thirdly, we will try to obtain the full text of these included articles, then further screening will be conducted by the same 2 reviewers to select eligible articles carefully. Meanwhile, the reason for their exclusion should be recorded. After that, to ensure the consistency of selection, the two reviewers will cross-check the results. During this period, a third senior reviewer (Shifeng Deng) will be invited to assist in the final judgment on the controversial articles. The specific selection process is shown in Figure 1.

In the next step, an information data extraction table will be established in Microsoft Excel 2016 to extract data and examine the consistency of information. The main content of data extraction will include first author, nationality, publication year, participant characteristics (gender, mean age, number of subjects, number of stroke episodes, stage of stroke, diagnostic criteria, ischemia or hemorrhage), methodological design (randomization, blind method, allocation concealment, bias risk), interventions (treatment measures, frequency, and course), comparator and outcomes (primary and secondary outcomes, drop-out cases or adverse events).

### 2.6 Dealing with missing data

When the data we need is incomplete, we will try to contact the author via email. In case of missing data are unreachable, intention-to-treat (ITT) analysis will be conducted.

### 2.7 Risk of bias assessment

The bias risk of all the included studies will be assessed by 2 trained researchers under the guidelines of Cochrane “Risk of bias “ assessment tool,^[32]^ which includes seven aspects: random sequence generation, assignment concealment, blinding of participants and personnel, blinding of outcome assessors, the integrity of outcome data, selective reporting and other sources of bias. In case of disagreement about the assessment, a third senior reviewer will be asked for arbitration.^[33]^ After that, the result will be shown in the “risk of bias” table.

### 2.8 Data synthesis and statistical methods

At firstly, a conventional meta-analysis will be conducted by Revman 5.3 software for the direct comparison results extracted from the literature. Next, a network meta-analysis based on the random effect model will be performed by R software 3.6.1 and WinBUGS 1.4.3 for the results of the indirect comparison. Under a Bayesian framework, the Markov chain Monte Carlo algorithm will be used to process the extracted data.^[34]^ Some details of preset parameters: 4 chains are used for simulation analysis, the step size is set to 10, the number of annealing times is set to 20,000, while the number of iterations is set to 50,000. In our study, the results of continuous variable (e.g., Fugl-Meyer Assessment score, Barthel Index) will be expressed by mean difference (MD) with 95% confidence interval; the effects of count data (e.g., adverse events, drop-out cases) will be calculated with the odds ratio (OR) and 95% confidence interval. Furthermore, we will calculate the surface under the cumulative ranking curve to forecast the possible ranking order of optimal acupuncture intervention time-point in stroke, in other words, which stage of stroke (e.g., hyperacute, acute, subacute, convalescent). Given that surface under the cumulative ranking curve values range from zero to one, the higher the value, the intervention time-point is considered to be better.^[35]^

### 2.9 Assessment of heterogeneity

The heterogeneity of the included studies will be checked by Stata 13.0 before the combination of effect size. In our study, we will discuss whether the characteristics of participants, interventions, and outcomes across all eligible studies are similar enough to each other. When clinical or methodological heterogeneity exists, statistical heterogeneity within each paired comparison will be evaluated by the I^2^ index. However, substantial heterogeneity will be regarded only if the I^2^ is above 50%.

### 2.10 Assessment of inconsistency, subgroup and sensitivity

In network meta-analysis, divergences among different kinds of evidence could be the main source of inconsistency. Thus, the loop inconsistency assessment of both direct and indirect evidence is indispensable. If necessary, we will determine the location of inconsistency with the help of the node-split method.

When considerable heterogeneity is found, subgroup analysis will be conducted based on the probable sources of heterogeneity (e.g., the duration of treatment, number of stroke episodes, age, or research quality).

Given that various levels of the methodological quality of studies could tend to affect the final result, we will conduct sensitivity analysis by eliminating literature with a high risk of bias.

### 2.11 Assessment of publication bias

First of all, to minimize the potential impact owing to publication bias, we will totally obey the above rules of literature selection. If the studies included are adequate (n≥10), a comparison-adjusted funnel plot will be used to assess the risk of publication bias visually.

### 2.12 Grading the quality of evidence

To grade the quality of each evidence, 2 reviewers will conduct the assessment separately according to the GRADE Working Group approach.^[36]^ Correspondingly, there are 4 quality levels: high, medium, low, and very low. When disagreement appears, a third one will be consulted.

## 3 Discussion

There are a significant number of patients with motor dysfunction and impairment of activities of daily living after stroke worldwide.^[3]^ Acupuncture therapy has been widely used in East Asian countries, especially in China, on patients with stroke as a kind of complementary and alternative therapy for many years.^[37]^ With more attention have been paid to the treatment and prognosis of cerebral stroke, an obvious increase in clinical and experimental studies of acupuncture in stroke has shown in recent years.^[38]^ However, most studies tend to focus on what kind of acupuncture therapy is more effective and the mechanism of it. In fact, what is the optimal acupuncture intervention time-point on patients after stroke still reminds an important problem, which confuses clinical choices. Therefore, it is easy to explain why the intervention time-point of acupuncture in stroke is not uniform in different clinical studies. During the preliminary work, we have noticed that the decision of using acupuncture on which stage of stroke (e.g., the hyperacute stage of stroke, the acute stage of stroke, the subacute stage of stroke, the convalescent stage of stroke) differs among different doctors and researchers. Some studies have been carried out in this field.^[39,40]^ However, network meta-analysis, recognized as a more valuable tool, can integrate direct and indirect comparisons across multi-dimensions.^[41]^ To our knowledge, it will be the first attempt in this respect.

However, there are some potential limitations that may affect the drawn conclusion in this study, such as the selection bias resulting from the limitation of languages, the difference of acupoint selections and acupuncture techniques, the difference of duration of intervention. Despite this, we do believe that the study results will help to figure out the optimal intervention time-point of acupuncture in the treatment of stroke, meanwhile, provide further suggestions for clinical practice or guidelines.

## Author contributions

**Conceptualization:** Yue Zhuo and Hong Zhang

**Data curation:** Yue Zhuo, Shifeng Deng, and Boyu Wu

**Methodology:** Yue Zhuo, Ming Xu, Shifeng Deng, Yuchen Zhang, Xiaoye Lu

**Formal analysis:** Yue Zhuo, Boyu Wu, and Ming Xu

**Supervision:** Hong Zhang

**Writing – original draft:** Yue Zhuo

**Writing – review & editing:** Yue Zhuo, Hong Zhang

## Correction

The number of the funded project for the Integrated Traditional Chinese and Western Medicine Open Fund, No.2020ZXYJH37, was originally omitted and has since been added.
